# Supplementary material for: Automated generation of gene-edited CAR T cells at clinical scale
Source: Mol Ther Methods Clin Dev. 2020 Dec 25;20:379–88. doi: 10.1016/j.omtm.2020.12.008 (PMC7848723; doi:10.1016/j.omtm.2020.12.008)
Supplement: Document S1. Figures S1–S4 [file mmc1.pdf]

OMTM, Volume *20*

Supplemental Information

Automated generation of gene-edited CAR T cells at clinical scale

Jamal Alzubi, Dominik Lock, Manuel Rhiel, Sabrina Schmitz, Stefan Wild, Claudio Mussolino, Markus Hildenbeutel, Caroline Brandes, Julia Rositzka, Simon Lennartz, Simone A. Haas, Kay O. Chmielewski, Thomas Schaser, Andrew Kaiser, Toni Cathomen, and Tatjana I. Cornu

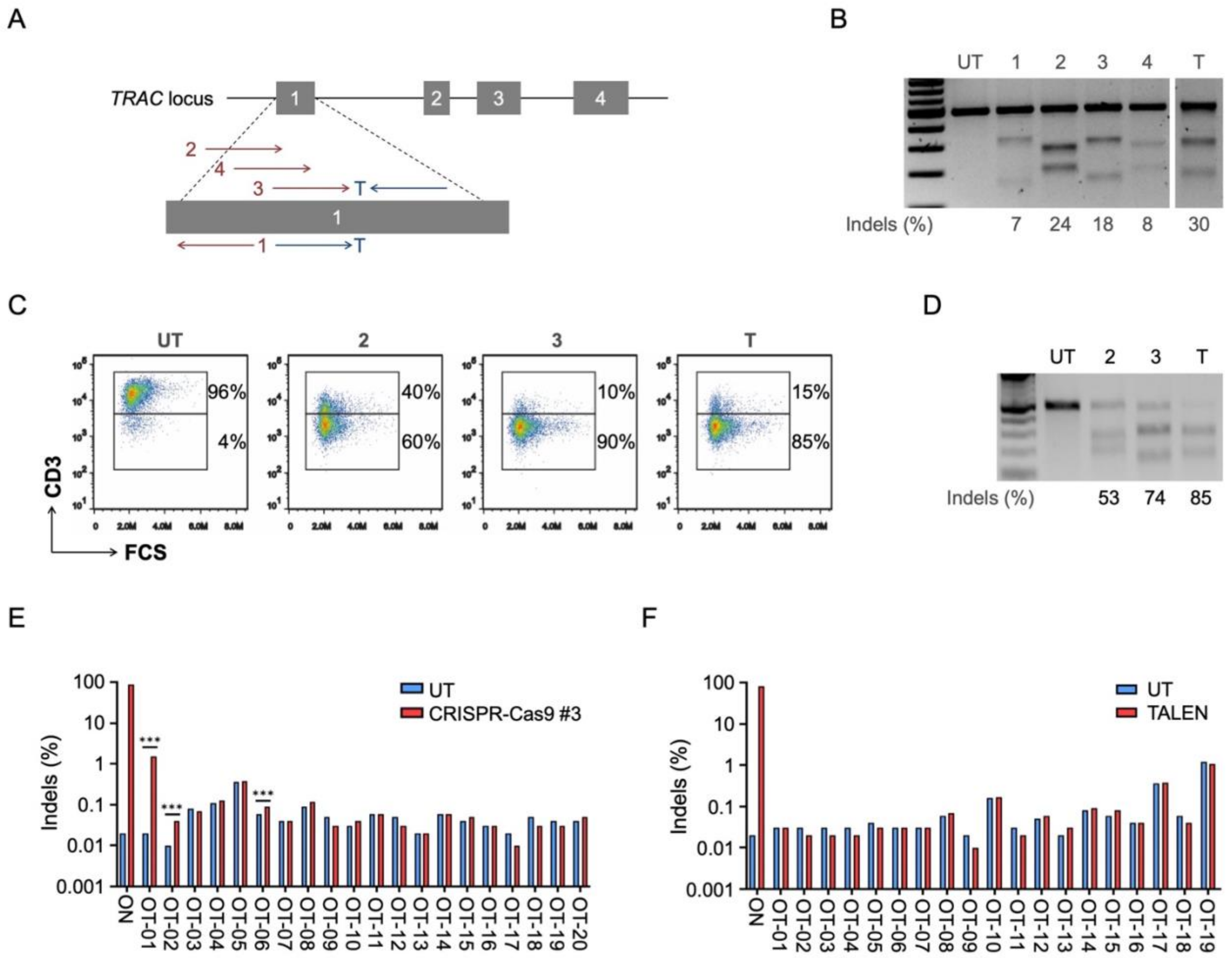

Figure S1. Characterization of *TRAC*-targeting designer nucleases. (A) Target sites in *TRAC* locus. Shown are the target sites in *TRAC* exon 1 of all designer nucleases. Red arrows denote CRISPR-Cas9 target sites #1-4, blue arrows represent binding half-sites of the TALEN (T). **(B)** Activity of designer nucleases in U2OS cells. U2OS cells transfected with designer nuclease expression plasmids (#1-4, T) were subjected to T7E1 assay. Percentages of cleaved products are indicated. Shown are results from one experiment. **(C)** Phenotyping of TALEN-edited T cells. Primary T cells nucleofected with either CRISPR-Cas9 ribonucleoprotein (RNP) complexes (#2-3) or TALEN-encoding mRNA (T) were subjected to flow cytometry. Shown are fractions of CD3-positive or CD3-negative cells harvested 7 days post-nucleofection. **(D)** Genotyping of TALEN-edited T cells. Primary T cells nucleofected with RNPs (#2-3) or TALEN-mRNA (T) were subjected to T7E1 assay. Percentages of cleaved products are indicated. Shown are results from one experiment. **(E-F)** Specificity profiles of *TRAC*-targeting designer nucleases. Primary T cells nucleofected with RNP (E) or TALEN mRNA (F) were subjected to targeted amplicon sequencing. Indel frequencies at the *TRAC* on-target site (ON) and potential off-target (OT) sites are indicated in comparison to untreated (UT) T cells. Shown are results from one donor. *** $p \leq 0.001$; Indels, insertion/deletion mutations.

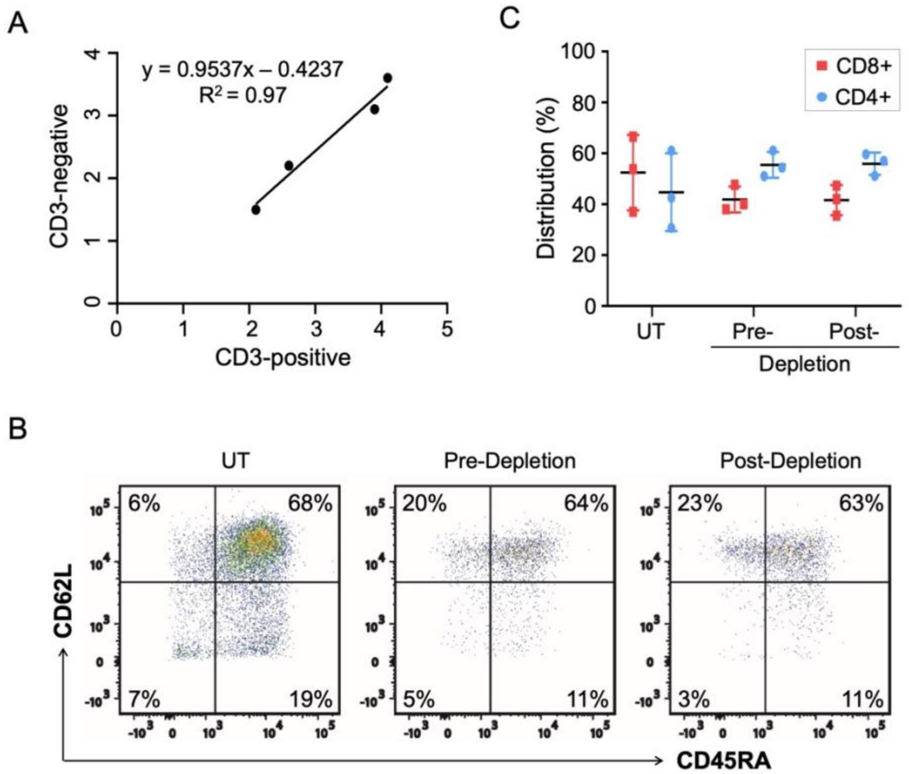

Figure S2. Quality assessment of *TRAC*-edited CAR T cells. (A) Comparison of cell division rates. Shown are the numbers of cell divisions for CD3-negative vs. CD3-positive cells determined at different time points during the manufacturing process (days 6, 7, 10, 12). Data were collected from all three manufacturing runs. **(B)** T cell phenotype analysis. T cell subsets were determined by assessing the expression of CD62L and CD45RA by flow cytometry. Edited CAR T cells were pre-gated for CD3 expression for UT, or CAR expression for the fraction of pre-depleted and post-depleted CAR T cells (not shown). Representative plots of one run are shown. **(C)** CD4 to CD8 ratio. Engineered T cells of three independent runs were harvested at indicated processing steps and CD4:CD8 ratio determined by flow cytometry.

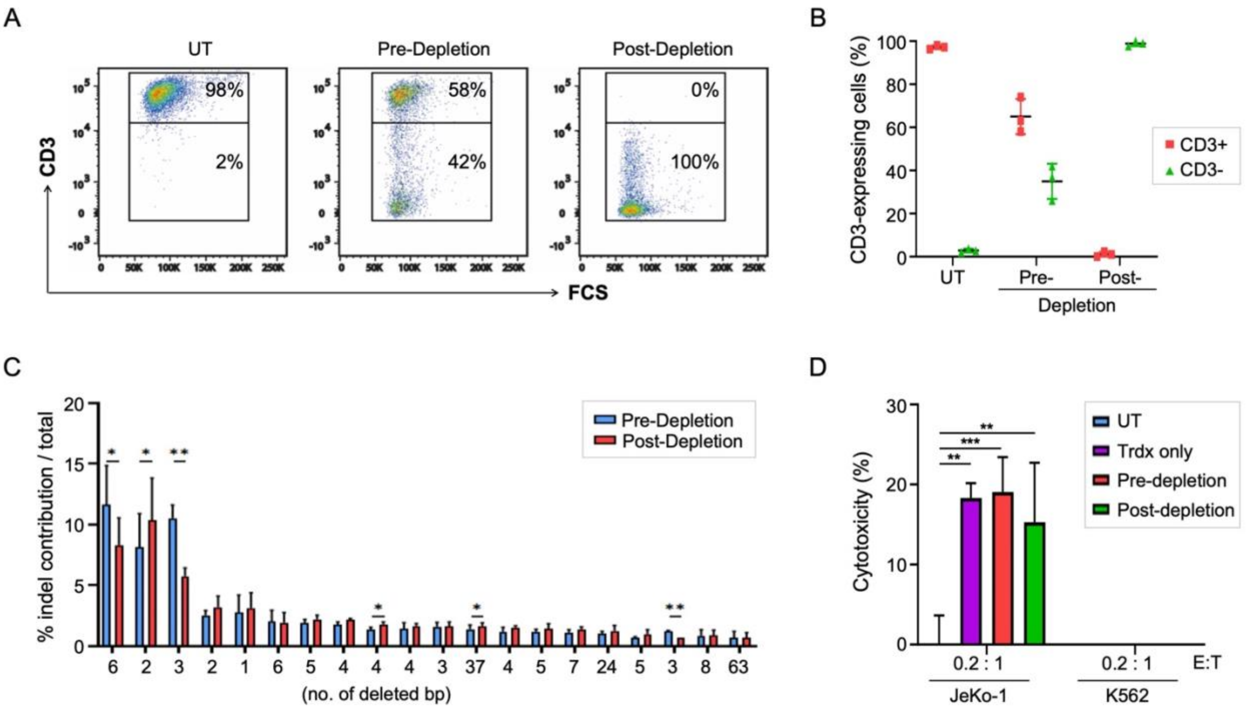

Figure S3. Characterization of *TRAC*-edited CAR T cells. (A) Phenotypic analysis of *TRAC* knockout. Engineered T cells were harvested at indicated processing steps. *TRAC* disruption was evaluated by flow cytometry analysis of CD3 expression. Representative plots of one run are shown. **(B)** Synopsis of three manufacturing runs. Shown are the fractions of CD3-positive and CD3-negative cells at indicated processing steps. **(C)** Indel distribution. Engineered T cells of pre and post-depletion steps were subjected to targeted amplicon sequencing of the *TRAC* locus. Shown are results of three manufacturing runs. Numbers on x-axis indicate the number of deleted bp. Indel, insertion/deletion; UT, untreated T cells. **(D)** Cytolytic activity. CAR T cells from indicated groups were co-cultured at an E:T ratio of 0.2:1 with CD19-positive JeKo-1 cells or CD19-negative K562 cells. Cytotoxicity was determined by flow cytometry. Shown are results of two or three independent runs, each in duplicate. UT, untreated T cells; Trdx only, CAR T cells retrieved directly after lentiviral transduction; Pre-depletion, CAR T cells harvested before TCRα/β depletion; Post-depletion, CAR T cells harvested after TCRα/β depletion.

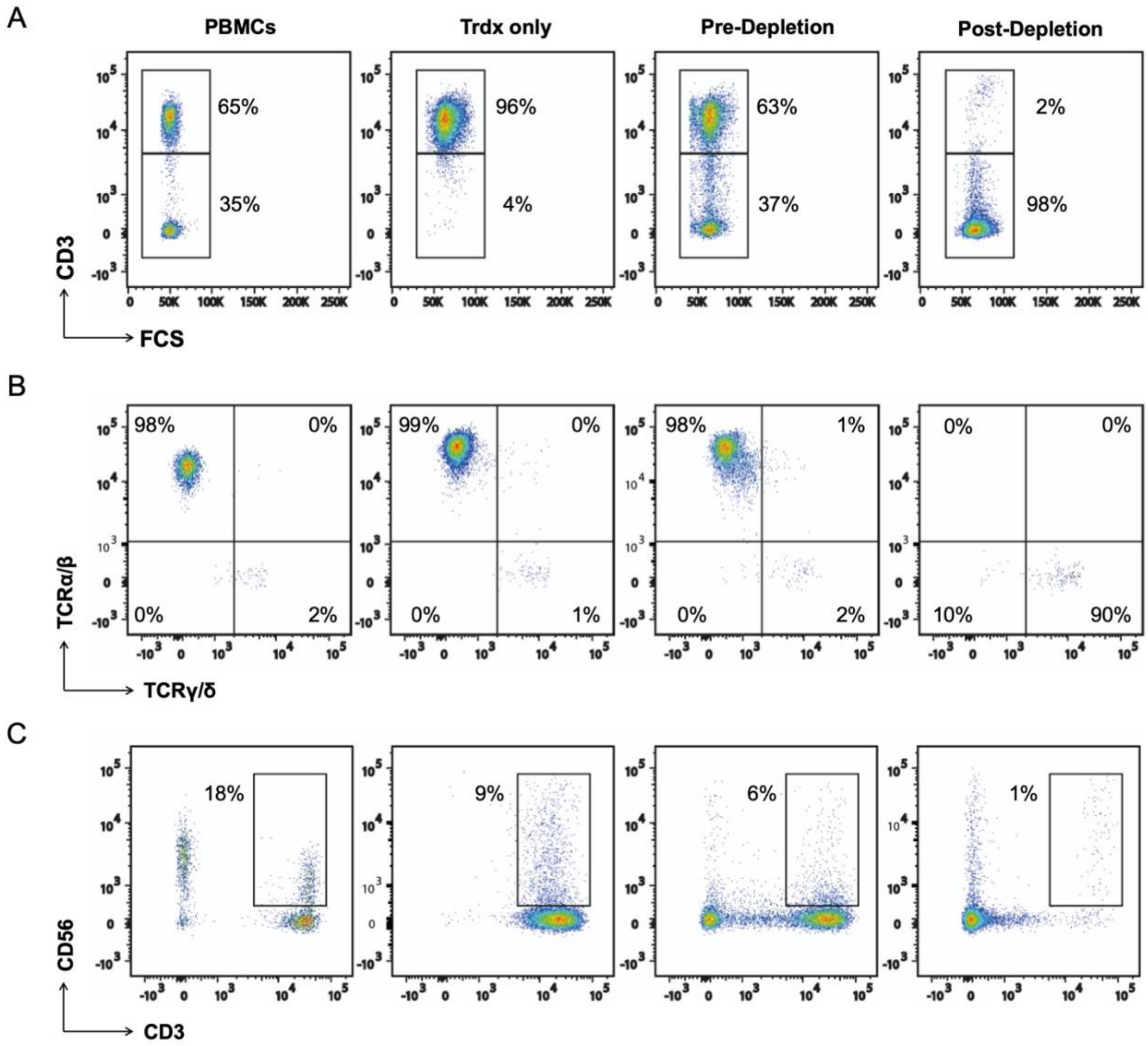

Figure S4. Characterization of final CAR T cell product. Engineered T cells were harvested at indicated processing steps and characterized in terms of **(A)** CD3 expression, **(B)** TCRα/β and TCRγ/δ expression, and **(C)** CD56 expression to identify the presence of NK and NKT cells. Shown are results of one representative run. PBMCs, peripheral blood mononuclear cells; Trdx only, T cells transduced with CD19-CAR encoding lentiviral particles only.
