# Supplementary material for: Automated generation of gene-edited CAR T cells at clinical scale
Source: Mol Ther Methods Clin Dev. 2020 Dec 25;20:379–88. doi: 10.1016/j.omtm.2020.12.008 (PMC7848723; doi:10.1016/j.omtm.2020.12.008)
Supplement: Document S2. Article plus supplemental information [file mmc2.pdf]

Molecular Therapy
Methods & Clinical Development
Original Article

Automated generation of gene-edited CAR T cells at clinical scale

Jamal Alzubi,[1,2,5] Dominik Lock,[3,5] Manuel Rhiel,[1,2] Sabrina Schmitz,[3] Stefan Wild,[3] Claudio Mussolino,[1,2,4] Markus Hildenbeutel,[1,2] Caroline Brandes,[3] Julia Rositzka,[1,2] Simon Lennartz,[3] Simone A. Haas,[1,2] Kay O. Chmielewski,[1,2] Thomas Schaser,[3] Andrew Kaiser,[3] Toni Cathomen,[1,2,4] and Tatjana I. Cornu[1,2,4]

[1]Institute for Transfusion Medicine and Gene Therapy, Medical Center - University of Freiburg, Freiburg, Germany; [2]Center for Chronic Immunodeficiency, Medical Center - University of Freiburg, Freiburg, Germany; [3]Miltenyi Biotec B.V. & Co. KG, Bergisch Gladbach, Germany; [4]Faculty of Medicine, University of Freiburg, Freiburg, Germany

The potential of adoptive cell therapy can be extended when combined with genome editing. However, variation in the quality of the starting material and the different manufacturing steps are associated with production failure and product contamination. Here, we present an automated T cell engineering process to produce off-the-shelf chimeric antigen receptor (CAR) T cells on an extended CliniMACS Prodigy platform containing an in-line electroporation unit. This setup was used to combine lentiviral delivery of a CD19-targeting CAR with transfer of mRNA encoding a *TRAC* locus-targeting transcription activator-like effector nuclease (TALEN). In three runs at clinical scale, the T cell receptor (TCR) alpha chain encoding *TRAC* locus was disrupted in >35% of cells with high cell viability (>90%) and no detectable off-target activity. A final negative selection step allowed the generation of TCRα/β-free CAR T cells with >99.5% purity. These CAR T cells proliferated well, maintained a T cell memory phenotype, eliminated CD19-positive tumor cells, and released the expected cytokines when exposed to B cell leukemia cells. In conclusion, we established an automated, good manufacturing practice (GMP)-compliant process that integrates lentiviral transduction with electroporation of TALEN mRNA to produce functional TCRα/β-free CAR19 T cells at clinical scale.

INTRODUCTION

A successful concept to treat hematological cancers is based on the genetic engineering of T cells to express chimeric antigen receptors (CARs). As opposed to the natural T cell receptor (TCR), CARs bind tumor antigens or tumor-associated antigens in a human leukocyte antigen (HLA)-independent manner. Anti-CD19 CAR T cells have shown remarkable success in treating CD19-expressing B cell malignancies, with the first two CAR T cell products (Kymriah, Yescarta) approved by the FDA and EMA in 2017 or 2018, respectively. The generation of CAR T cells typically involves the delivery of a CAR transgene into autologous T cells using (semi-)randomly integrating viral or non-viral vector systems.[1] However, the generation of CAR T cells from autologous sources is highly dependent on the quality of the starting T cell apheresis material. In fact, 9% of the patients enrolled in the pivotal trial entailing Kymriah did not receive their CAR T cell products due to manufacturing failure.[2] In order to overcome these limitations, “off-the-shelf” or “universal” CAR T cell products were generated by editing in allogeneic T cells the *TRAC* or *TRBC* genes encoding the TCR α or β chain, respectively.[1,3–8] All major classes of designer nucleases, zinc-finger nucleases (ZFNs),[9,10] transcription activator-like effector nucleases (TALENs)[11] and CRISPR-Cas9 nucleases,[5] have been used for this purpose.[12] Knockout of either locus will disrupt TCRα/β pairing and hence eliminate surface expression of the TCR/CD3 complex. Absence of TCR in turn alleviates autoreactivity or alloreactivity of the engineered T cells.[10] Recently, two infants suffering from relapsed B cell acute lymphoblastic leukemia (B-ALL) were successfully treated with gene-edited, off-the-shelf anti-CD19 CAR T cell products.[13] An additional modification introduced by TALENs included the disruption of the *CD52* gene, which encodes a surface marker targetable with alemtuzumab. CD52 knockout cells are resistant to alemtuzumab and can therefore be combined with this antibody-based treatment of leukemia.[13]

Generation of CAR T cells in compliance with good manufacturing practice (GMP) is a complex multistep process including enrichment, activation, lentiviral transduction, and expansion. This is even more pronounced when combined with an additional electroporation step to deliver gene-editing tools, such as designer nucleases. While several GMP-compliant protocols to engineer CAR T cells using viral vectors or mRNA transfer have been described,[14–16] the automation of the manufacturing processes in a fully closed system is attractive to reduce risks of contaminations and simplify manufacturing at clinical scale in a highly reproducible manner.[17–19]

Here, we aimed at generating an off-the-shelf CD19-targeting CAR T cell product in an automated process at clinical scale. To this

Received 27 October 2020; accepted 21 December 2020;
https://doi.org/10.1016/j.omtm.2020.12.008.

[5]These authors contributed equally

Correspondence: Toni Cathomen, Institute for Transfusion Medicine and Gene Therapy, Medical Center - University of Freiburg, Freiburg, Germany.
E-mail: toni.cathomen@uniklinik-freiburg.de

This is an open access article under the CC BY license (http://creativecommons.org/licenses/by/4.0/).

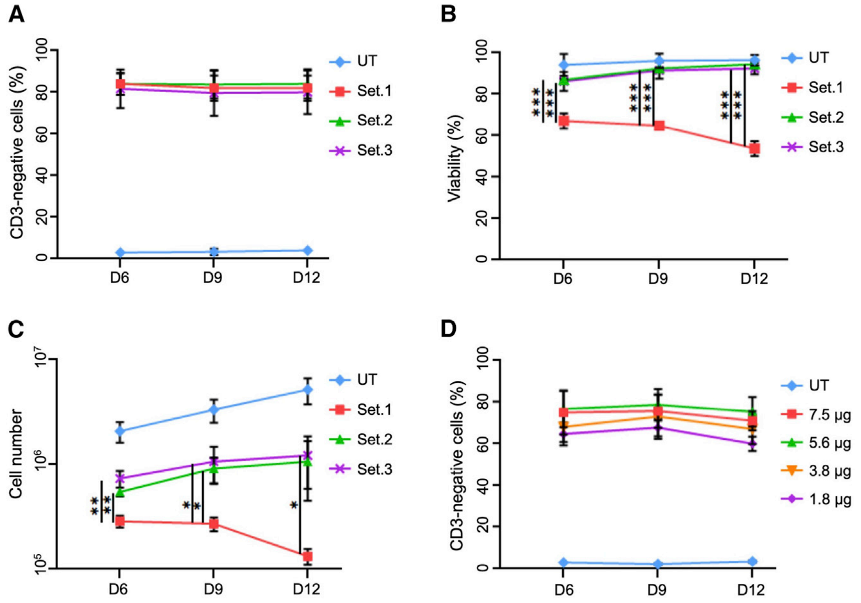

Figure 1. Optimization of electroporation conditions

(A) Disruption of *TRAC* locus. Activated T cells were electroporated with TALEN-encoding mRNA (7.5 μg) using three different settings. Edited T cells were harvested at indicated time points and extent of T cell receptor knockout quantified by measuring CD3 expression. (B and C) Cell viability and cell proliferation. Electroporation-associated toxicity (B) and cell numbers (C) were determined using an automated cell counter (NucleoCounter). (D) Titration of the *TRAC*-targeting TALEN mRNA. Following electroporation with the indicated amounts of TALEN RNA (setting 3), cells were harvested and extent of T cell receptor knockout quantified by measuring CD3 expression. $^{*}p \leq 0.05$, $^{**}p \leq 0.01$, $^{***}p \leq 0.001$. UT, untreated T cells; Set.X, electroporation settings (see Table 1).

end, we enabled reproducible GMP-compliant manufacturing of gene-edited T cells on a CliniMACS Prodigy connected to an in-line electroporator. We used lentiviral transduction to deliver a 2nd-generation anti-CD19 CAR, composed of a FMC63-derived single chain variable fragment (scFv), a CD8-derived hinge region, a TNFRSF19-derived transmembrane domain linked to the 4-1BB co-stimulatory domain, and a CD3ζ signaling domain.[20] This was combined with electroporation of the T cells to transfer mRNA encoding a *TRAC* targeting TALEN. We report on cellular composition, T cell phenotype, as well as *TRAC* knockout efficacy and demonstrate that a >99.5% TCRα/β-free CAR T cell product can be generated with high quality and conservation of its cytolytic potency.

RESULTS

Identifying the best designer nuclease to target the *TRAC* locus

In order to disrupt the *TRAC* locus, a TALEN and four CRISPR-Cas9 nucleases were designed to target the first exon of the constant region of *TRAC* (Figure S1A). To identify the best-performing programmable nuclease, they were initially screened by transfecting human U2OS cells with the respective expression plasmids followed by applying the mismatch-sensitive T7 endonuclease 1 (T7E1) assay on PCR amplicons encompassing the target site (Figure S1B). The most promising three candidates were then evaluated in primary T cells. To this end, T cells were activated for 3 days before being nucleofected with the indicated CRISPR-Cas9 ribonucleoprotein (RNP) complexes or with TALEN-encoding mRNA. After 7 days, cells were phenotyped by monitoring CD3 expression (Figure S1C) or genotyped by T7E1 assay (Figure S1D). Upon delivery of CRISPR-Cas9 nuclease #3 or TALEN, >85% of T cells were CD3 negative and >74% of the *TRAC* alleles were disrupted. As the specificity of the developed nucleases is important in terms of clinical safety, we evaluated the activity and specificity profiles of the two designer nucleases by next-generation targeted amplicon sequencing (Amp-seq) of the on-target site and the top predicted off-target sites (Figures S1E and S1F). Amp-seq confirmed that >80% of on-target alleles contained insertion/deletion mutations (indels) with either customized nuclease. However, while the CRISPR-Cas9 nuclease showed significant activity at three off-target sites, no significant off-target activity was detected upon TALEN expression. In conclusion, we were able to design a highly active and specific TALEN to target the *TRAC* locus that was used for all subsequent experiments.

Optimizing the electroporation conditions for delivery of mRNA

To optimize the best conditions for transferring mRNA to T cells, we performed small-scale electroporations on the CliniMACS Prodigy-connected electroporator using a test cuvette adaptor in order to identify the best electroporation settings and the optimal concentration of *TRAC*-targeting TALEN mRNA. For this purpose, activated T cells were electroporated with 7.5 μg of each TALEN mRNA at day 3 post-activation using 3 different settings (Figure 1; Table 1). Afterward, edited T cells were expanded up to 12 days, and the knockout efficiency, viability, and total cell number were monitored every 3 days post-electroporation. Independently of the settings used, we achieved high and stable TCR knockout efficiencies, as indicated by >80% CD3-negative cells (Figure 1A). We observed that T cells electroporated with setting 1 showed significant electroporation-associated toxicity (Figure 1B), which in turn was associated with impaired expansion capacity of those cells (Figure 1C). T cells electroporated with settings 2 or 3, on the other hand, recovered well after electroporation, as indicated by >90% cell viability (Figure 1B) and ensuing expansion (Figure 1C). Based on these results, we chose setting 3 to be used in the subsequent experiments. Next, we evaluated the window of activity of our TALEN in order to identify the optimal amounts of TALEN mRNA to be transferred to cells. We monitored TALEN activity by quantifying the fraction of CD3-negative cells. 70%–80% of T cells were CD3 negative when using 3.8–7.5 μg of each TALEN mRNA (Figure 1D). Together, these experiments confirmed the possibility to develop highly active and specific

Table 1. Electroporator settings

Setting	Prodigy electroporator
Set.1	Pulse 1, 950 V, 120-μs burst/flip—interval; 8-μs pause
	Pulse 2, 125 V, 23.000-μs burst—interval; 8-μs pause
Set.2	Pulse 1, 600 V, 120-μs burst/flip—interval; 8-μs pause
	Pulse 2, 400 V, 2.000-μs burst—interval; 8-μs pause
Set.3	Pulse 1, 600 V, 104-μs burst/flip—interval; 8-μs pause
	Pulse 2, 400 V, 2.000-μs burst—interval; 8-μs pause

TRAC-targeting TALEN, which can be scaled-up and used on the CliniMACS Prodigy platform in the form of mRNA with limited electroporation-associated toxicity.

Automated generation of TCRα/β-free CAR T cells at clinical scale

We established a process for the automated generation of TCRα/β-free CAR T cell product in a closed system and validated the protocol at two different sites with three clinical scale runs in total. The full automation process is described in detail in the Materials and methods (Figure 2A). In brief, on day 0, a fresh buffy coat or leukapheresis product was sterile welded to the tubing set to magnetically isolate CD4-positive and CD8-positive T cells that were subsequently activated using a nanomatrix-based anti-CD3/28 polyclonal T cell stimulator. On day 1, T cells were transduced with anti-CD19 CAR-encoding lentiviral particles. On day 3, T cells were electroporated with *TRAC*-targeting TALEN mRNA and expanded for up to 13 days in medium supplemented with interleukin (IL)-7 (IL-7) and IL-15. During the process, medium was replaced every other day until day of harvest. During a subsequent TCRα/β-depletion step, 50% of the harvested cellular product was transferred into a second tubing set to remove remaining TCRα/β-expressing T cells. The final cellular T cell products, pre- and post-depleted, were formulated and frozen until further use. Over the entire period of the manufacturing process, quality control (QC) pouches enabled in-process controls for assessment of cell count, viability, cellular composition, phenotype, and *TRAC* knockout efficiencies. The manufacturing process started with 2×10^8 selected T cells that exponentially expanded starting 24 h after electroporation and finally resulted in comparable cell numbers of approximately 2×10^9 total T cells (Figure 2B). As expected, due to an electroporation-associated toxicity, we observed a decline in viability directly after the electroporation; however, the cells quickly recovered within the following 48 h with >95% viable cells until the end of the entire manufacturing process (Figure 2C). Furthermore, CD3-negative and CD3-positive cells divided at comparable rates (Figure S2A), indicating that *TRAC* editing had no detectable impact on cell growth throughout the manufacturing process in the Prodigy. Additionally, we evaluated the cellular composition during the different stages of the manufacturing process. Upon the first selection step, the CD4/8 fraction consisted mainly of T cells (∼80%) with some monocytes due to low CD4 expression and some natural killer (NK) cells due to low CD8 expression (Figure 2D). Importantly, following CD4/8 selection and during the entire process, we did not observe any enrichment of B cells (Figure 2D). At the end of the culture, residual monocytes were lost; the final product contained >99% T cells with a favorable memory T cell phenotype (Figures 2E and S2B) and the expected 1:1 ratio of CD4 to CD8 T cells (Figure S2C).[18–20] Overall, this demonstrates the possibility to combine transduction and electroporation in one closed and automated process yielding a therapeutic dose of viable engineered T cells.

Gene-editing efficiency and off-target analysis

Next, we evaluated *TRAC* gene disruption at both phenotypic and genotypic levels. At the end of the expansion phase, ∼35% of cells were TCRα/β-negative (pre-depletion), which was increased to almost 100% TCRα/β-negative cells after a negative selection (post-depletion) step (Figures 3A and 3B). These results were in line with the observed reduction in CD3-positive cells (Figures S3A and S3B) and genotyping by Amp-seq, which confirmed indel frequencies of 35%–42% at the on-target site before selection and almost 100% after selection, respectively (Figure 3C). Furthermore, we monitored the indel distribution over the entire manufacturing process for two runs and did not observe any enrichment or loss of any mutation type throughout the manufacturing process (Figure 3D), suggesting the absence of clonal selection. The most common indels observed were 6-bp, 2-bp, and 3-bp deletions, respectively, which probably reflect favored DNA repair outcomes due to the presence of microhomologies (data not shown). Of note, when we analyzed the indel patterns in the cell population before and after TCRα/β depletion, we observed a significant drop in the frequencies of some in-frame deletion variants (Figure S3C), probably as a result of the elimination of edited CAR T cells that still expressed TCRα/β despite lacking 1 or 2 amino acids. A detailed analysis of the final depletion step with unedited CAR T cells confirmed its efficacy to remove TCRα/β-positive T cells. The remaining CD3-positive cells in the post-depletion fraction were either γ/δ T cells or NK T cells (Figure S4). In summary, our automated process combined efficient transduction of T cells with efficacious and genetically stable TCRα/β knockout in order to generate TCRα/β-free CD19-directed CAR T cells with >99.5% purity.

Functional characterization of the TCRα/β-free CAR T cells

Upon transduction, the fraction of CAR-positive cells remained stable during the whole manufacturing process and ranged from 40%–69% during the 3 runs (Figure 4A). These results validate stable CAR expression and confirm that neither gene editing nor the TCR depletion step negatively affected the frequency of CAR-expressing T cells in the final cell product. Potency of the final product was assessed by determining the cytotoxic activity and the cytokine release profile of the engineered CAR T cells. As target cells, we used the CD19-positive mantle cell lymphoma line Jeko-1, the CD19-positive ALL line Nalm6, and CD19-negative K562 cells as a control (Figure 4B). To assess the impact of the TCR complex in the functionality of the CAR T cells, we compared TCRα/β-free CAR T cells with CAR T cells that were only transduced with lentiviral vector but not edited (Trdx only). As a further sample, cells from the pre-depletion fraction, containing TCRα/β-positive and TCRα/β-negative CAR

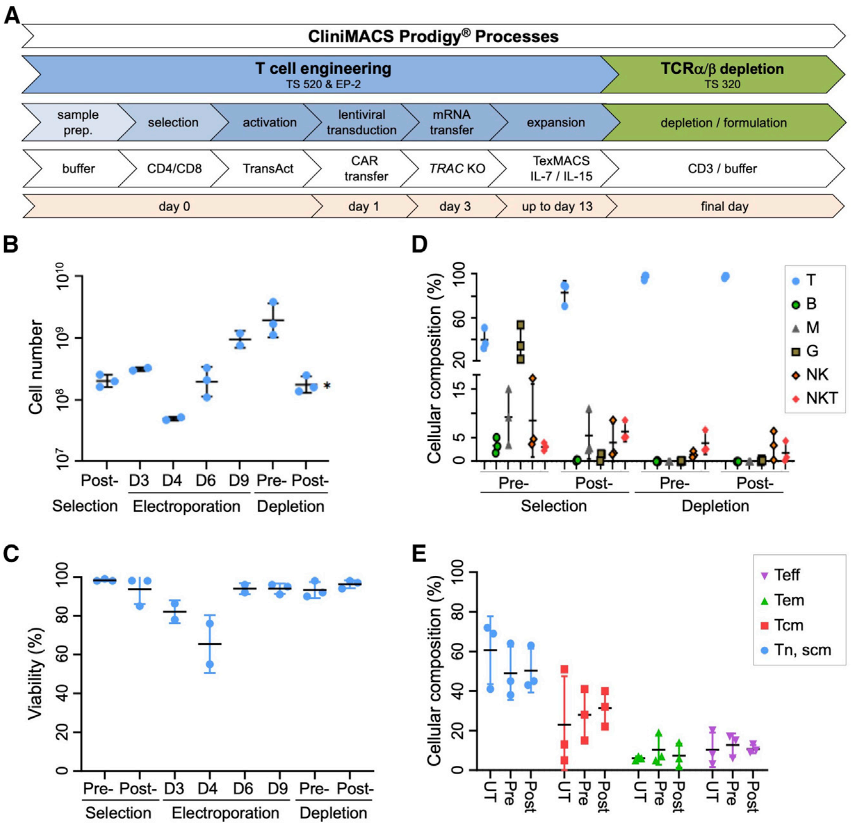

Figure 2. Automated generation of TCRα/β-free CAR T cells

(A) Schematic overview of the automated process on the CliniMACS Prodigy platform. Day 0, T cell selection and activation. Day 1, T cell transduction with CD19-CAR encoding lentiviral particles. Day 3, electroporation of transduced T cells with *TRAC*-targeting TALEN. Days 9–13, expansion of engineered T cells followed by TCRα/β depletion, harvesting, and cryopreservation. (B and C) Cell expansion and cell viability. Cell numbers (B) and cell viability (C) were determined using an automated cell counter (NucleoCounter). (D and E) Cellular composition. Representative samples were taken from the culture at indicated cell processing steps, and cellular composition was determined by flow cytometry (see Materials and methods). Shown are the single data points and the averages of three runs. T, T cells; B, B cells; M, monocytes; G, granulocytes; NK, natural killer cells; NKT, natural killer T cells; Tn/Tscm, T cell naive or T stem cell memory; Tcm, T cell central memory; Tem, T cell effector memory; Teff, T cell effector; UT, untreated T cells. *, the depletion process started with 50% of T cells from the pre-depletion step.

T cells, were assessed side-by-side with untreated T cells. The different samples were co-cultured with CD19-positive or -negative target cells at different effector-to-target (E:T) ratios before cytotoxicity was evaluated by determining killing of marked target cells (Figures 4C and S3D). The cytotoxic activities were comparable for all samples, i.e., all CAR-expressing cells eliminated approximately 60%–70% of CD19-positive Nalm6 cells at a 1:1 E:T ratio, 50% of CD19-positive Jeko-1 cells at a 1:1 E:T ratio (Figure 4C), and circa 20% of Jeko-1 cells at an E:T ratio of 0.2:1 (Figure S3D). No significant cytotoxic activity was observed when CAR T cells were co-cultured with K562 cells (Figures 4C and S2C). Supernatants from co-cultures of CAR T cells with Jeko-1 cells were used to quantify the cytokines secreted by activated CAR T cells (Figure 4D). Comparable cytokine levels (granulocyte macrophage colony-stimulating factor GM-CSF, interferon [IFN]-γ, IL-2, and tumor necrosis factor alpha [TNF-α]) were detected in all CAR T cell samples upon exposure to CD19-positive lymphoma cells. In summary, we were able to generate potent TCRα/β-free CAR T cells that showed similar *in vitro* functionality when compared to unedited CAR T cells for both cytotoxic potency and cytokine release levels.

DISCUSSION

A process enabling the dissemination of CAR T cell therapies to larger patient numbers should be robust, reproducible, scalable, and cost efficient, and it has to fulfill all regulatory requirements.[21] Furthermore, such a complex multistep procedure requires extensively trained personnel as well as a dedicated infrastructure.[20] To meet these demands, we established a protocol to manufacture a TCRα/β-free CAR T cell product in a closed, automated, GMP-compliant process on the CliniMACS Prodigy platform. To restrict T cell specificity, we combined lentiviral CAR transgene transfer with an in-line electroporation step to deliver a TALEN-encoding mRNA to knock out the *TRAC* locus. After a final TCRα/β depletion step, we achieved >99.5% TCRα/β-free CAR T cells. Analysis of the cellular composition and the T cell phenotype confirmed the manufacturing of a highly pure T cell product with a favorable memory T cell phenotype. Importantly, the TCRα/β-free CAR T cells were equally functional in *in vitro* assays when compared to the unedited CAR T cells in terms of cytotoxicity and cytokine release profile.

In-depth phenotypic characterization of the final cell product revealed the presence of some CD3-positive cells that did not express TCRα/β but rather the TCRγ/δ complex or NK cell markers. It was shown that the presence of NK cells and/or TCRγ/δ T cells may have a protective role against leukemia relapses and mediate graft-versus-leukemia effects.[22] A recent report of a relapse caused by leukemic B cells that were unintentionally transduced with the CAR expression vector underlines the importance of product purity.[23] Due to the selection of CD4/CD8 as initial process step as well as culture conditions favoring T cell outgrowth, potential B cell contaminations of the final T cell product can therefore be practically eliminated in our manufacturing process.

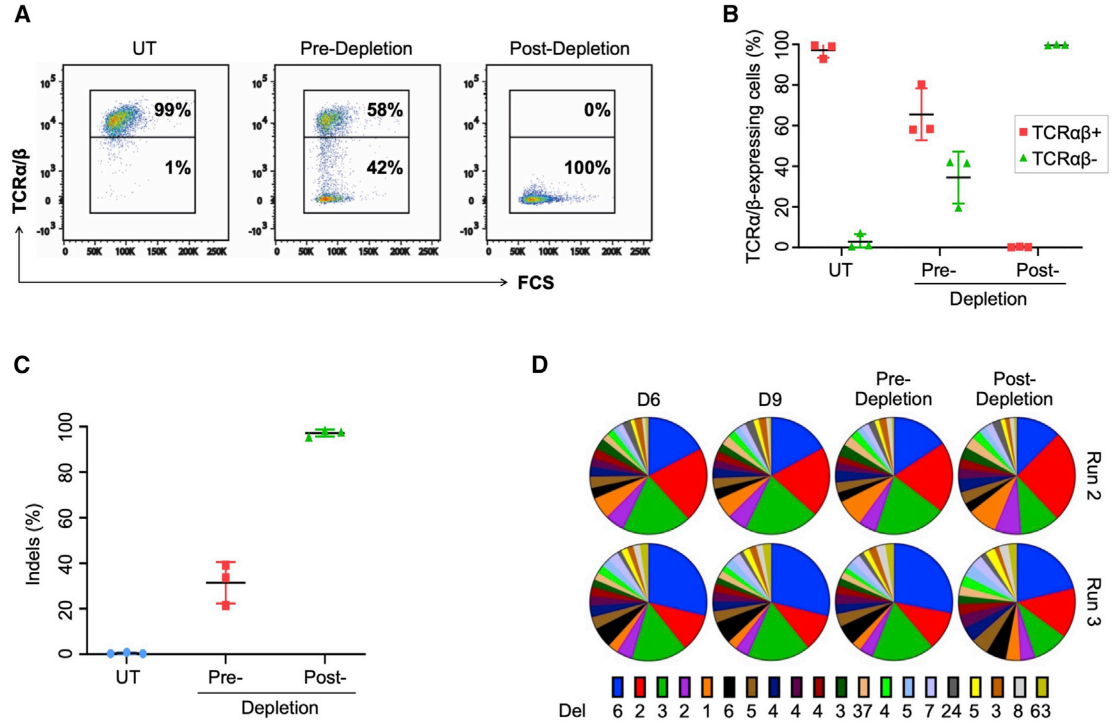

Figure 3. Evaluation of *TRAC* disruption efficiency

(A) Phenotypic analysis. Cells were harvested from pre- and post-depletion steps and T cell receptor (TCR) α/β knockout efficiency evaluated by flow cytometry. Representative plots from one run are shown. (B) Synopsis of three runs. Shown are the single data points and the average fractions of TCRα/β-positive or -negative cells from the pre- and post-depletion steps of three runs. (C) Genotypic analysis. Targeted amplicon sequencing of the *TRAC* locus was performed on TALEN-edited cells harvested at pre-and post-depleted steps. Shown are the single data points and the average indel frequencies of three runs. (D) Distribution of TALEN-induced deletions. Engineered T cells were harvested at indicated time points and subjected to targeted amplicon sequencing of the *TRAC* locus. The distributions of the induced deletions (Del) of two runs are shown as pie charts. Numbers indicate the number of deleted bp. Indels, insertions/deletions; UT, untreated T cells.

Off-the-shelf CAR T cells were generated by employing designer nucleases to target *TRAC* or *TRBC*.[3–8,11,12] Knockout of either gene prevents the expression of the TCRα/β complex, hence allowing the generation of an allogeneic cell product with restricted T cell specificity. Recently, it was reported that the lack of tonic TCR signaling may lead to lower persistence of TCR-free CAR T cells.[7,24] These observations, even if confirmed by others in the future, do not preclude the forthcoming application of TCRα/β-free CAR T cells. We believe that off-the-shelf CAR T cell products are, e.g., a highly attractive product for a bridge-to-transplant therapy, for patients that need instant therapeutic intervention because of high tumor burden or in case of manufacturing failure of the autologous product due to insufficient quality of the starting material.

The described process can be easily adapted to enable the transfer of CRISPR-Cas nucleases or to transduce cells with adeno-associated virus (AAV)-based vectors for targeted integration of the CAR cassette. Moreover, the Prodigy platform is amenable to more complex and/or multiplex genome-editing approaches. Two laboratories targeted the integration of a CD19-CAR into the *TRAC* locus using a CRISPR-Cas9 nuclease and an AAV-based vector to deliver the CAR.[4,5] This strategy knocks out the TCR and places CAR expression under control of the endogenous TCR promoter, which resulted in both an improved cytolytic potential as well as a reduced exhaustion of the CAR T cells.[5] Thus, one would not only manufacture an off-the-shelf CAR T product by knocking out TCRα/β but also reduce the potential risk of insertional oncogenesis when using (semi-)randomly integrating lentiviral particles. An exciting alternative to retroviral transduction is the use of *Sleeping Beauty* transposition using an electroporation-based protocol.[25] Here, the benefits in the manufacturing of CAR T cells comprise an increased cargo capacity as well as a close-to-random genomic integration pattern. Further efforts are focusing on the development of protocols allowing to manufacture autologous, fratricide-resistant CAR T cells required to treat defined T cell malignancies[26] or the knockout of checkpoint inhibitors as a powerful step toward the treatment of solid tumors.[27] Toward further simplifying the manufacturing process of off-the-shelf products, it is worth pointing out the strategy developed by Juillerat and colleagues,[8] who demonstrated that transient expression of an anti-CD3 CAR by mRNA transfer together with expression of *TRAC*-targeting TALEN induced self-elimination of the CD3-positive T cell fraction and hence generation of TCRα/β-free CAR T cells without the need of further selection. An interesting study by Straetemans et al.[14] described the manufacturing of TCRα/β-free T cells by expressing a defined

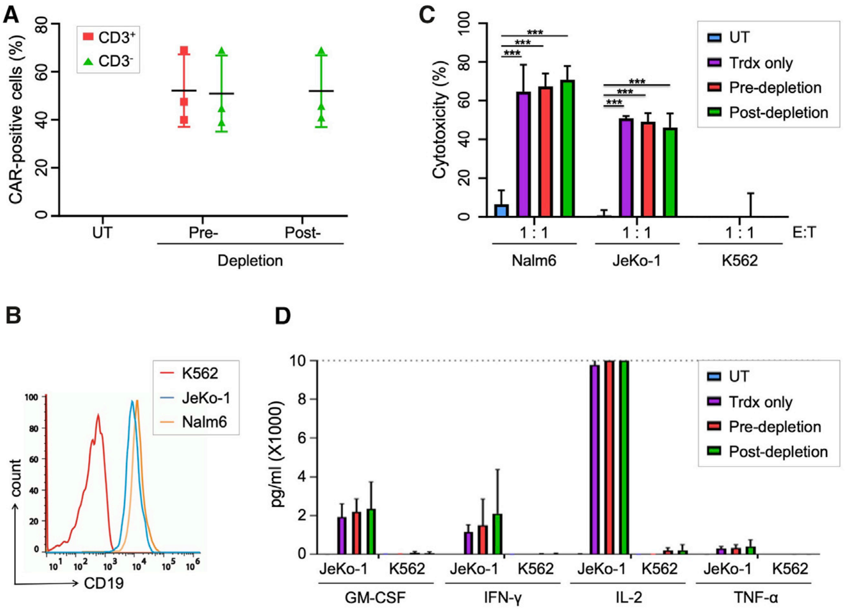

Figure 4. *In vitro* functionality of TCRα/β-free CAR T cells

(A) CAR expression. Edited CAR T cells were harvested at pre- and post-depletion steps, and the percentage of CAR-expressing cells in the CD3-positive or CD3-negative fraction determined by flow cytometry. (B) Characterization of target cells. Jeko-1, Nalm6, and K562 cells were stained with anti-CD19 antibody and expression levels assessed by flow cytometry. (C) Cytolytic activity. CAR T cells from indicated groups were co-cultured at a 1:1 E:T ratio with CD19-positive Jeko-1 cells, CD19-positive Nalm6 cells, or CD19-negative K562 cells. Cytotoxicity was determined by flow cytometry. (D) Cytokine release. The concentration of indicated cytokines was determined in supernatants of CAR T cells cultured with Jeko-1 cells at a 1:1 ratio. UT, untreated T cells; Trdx only, CAR T cells retrieved directly after lentiviral transduction; pre-depletion, CAR T cells harvested before TCRα/β depletion; post-depletion, CAR T cells harvested after TCRα/β depletion; GM-CSF, granulocyte/monocyte colony-stimulating factor, IFN-γ, interferon gamma; IL-2, interleukin-2; TNF-α, tumor necrosis factor alpha. *** $p \leq 0.001$.

high-affinity γ/δ-TCR. This TCR was introduced by retroviral transduction, and it was shown that overexpression of the γ/δ-TCR outcompeted the endogenous TCRα/β. The remaining TCRα/β-expressing cells were depleted in a GMP-compliant process using the CliniMACS system. Although the manufacturing protocol differs from ours and their starting material contained higher amounts of TCRα/β-expressing cells, the efficiencies of depletion were in line with our observations in the final product.

More recently, CAR integration into the *TRAC* locus was combined with concomitant targeted integration of an IL-12 transgene into the *PDCD1* or *IL2RA* loci, both of which code for immune cell activators.[28] The result was transient, antigen concentration-dependent IL-12P70 secretion, which increased cytotoxicity of these off-the-shelf CAR T cell product. Multiplex gene editing with two or more nucleases has the advantage of reducing the number of manufacturing steps. The simplicity and monomeric nature of the CRISPR-Cas9 platform certainly facilitates such multiplex approaches.[1,29] On the other hand, target site cleavage by TALENs is mediated by two TALEN subunits that form a heterodimer when assembling at the target site.[12] Multiplexing is therefore more challenging: the concomitant targeting of two loci already requires the transfer of four TALEN subunits, which—in theory—can form six different heterodimers and four homodimers in the cell, hence increasing the risk of off-target activity.[11] Hence, in any case, the final product must be carefully tested for off-target effects and take into account the higher risk of inducing chromosomal translocations due to concomitant cleavage of the genome at multiple sites.[4,11,29]

In general, to identify the optimal engineered nuclease to target a locus of choice, one must address both the activity and specificity profiles for every single target locus. Accordingly, we compared our *TRAC*-targeting designer nucleases side-by-side and identified a TALEN that combines high activity with high specificity. Any further combination with another customized nuclease needs reevaluation to determine the combined genotoxic risk.

While opening new therapeutic opportunities, the ability to edit genes through electroporation-based transfer of designer nucleases in a GMP-compliant setting will inevitably increase manufacturing costs for adoptive T cell therapies. Besides the initial expenses to acquire the platform, the additional spending on buffers and tubing accessory is neglectable when compared to the underlying T cell manufacturing process. A major cost driver is, however, the GMP-grade RNA required for gene editing. Similar to GMP-grade viral vectors, costs are likely to drop as manufacturing of such materials improves and batch sizes increase. Reduction in such costs of goods will be essential in order to further disseminate the use of transduction-based manufacturing protocols or non-viral gene-editing approaches.

In conclusion, our results demonstrated that *in vitro* functionality of gene-edited CAR T cells that were manufactured in an automated fashion were comparable to unedited CAR T cell controls. Such protocols will facilitate the production of off-the-shelf CAR T cell products and their successful clinical application.

MATERIALS AND METHODS

TRAC-targeting designer nucleases

The *TRAC* gene sequence was retrieved from the NCBI database. Designer nucleases were designed to cleave the first exon of the constant region of the *TRAC* gene. The TALEN pair was designed and generated as previously described.[30] The target sequence for the TALEN pair is 5′-TAGACATGAGGTCTATGGActtcaagagcaacAG

TGCTGTGGCCTGGAGCA-3′, where the 19-bp recognition sites for both TALEN (TALEN-L and TALEN-R) monomers (uppercase letters) are separated by a 13-bp spacer sequence (lowercase letters). CRISPR-Cas9 guide RNAs (gRNAs) were designed using the CHOP-CHOP web tool[31] to target the following sequences: CRISPR-Cas9 #1 (5′-TGGATTTAGAGTCTCTCAGC-3′), CRISPR-Cas9 #2 (5′-ACAA AACTGTGCTAGACATG-3′), CRISPR-Cas9 #3 (5′-CTTCAAGAG-CAACAGTGCTG-3′), and CRISPR-Cas9 #4 (5′-TGTGCTAGACAT-GAGGTCTA-3′). TALEN encoding plasmids including a T7 promoter were linearized and used as templates for mRNA production by *in vitro* transcription (IVT) using T7 RNA polymerase. The *in vitro* mRNA transcripts were purified using a RNeasy kit (QIAGEN), enzymatically capped, and polyadenylated. After DNase treatment, RNA concentrations were determined by measuring the absorbance at 260 nm using a nanodrop spectrophotometer (Thermo Fisher). The length of the *in vitro* transcripts and the polyadenylated mRNAs were monitored by Bioanalyzer electrophoreses on an RNA nano chip (Agilent). The gRNAs were ordered with the following chemical modifications: 2′-O-methyl analogs and 3′ phosphorothioate internucleotide linkage at the 5′ and 3′ terminal three bases (Synthego). SpCas9 protein was purchased (PNA Bio).

Assessment of *TRAC*-targeting designer nucleases

U2OS cells (identity confirmed by short tandem repeat genetic profiling; Eurofins) were cultured in Dulbecco's modified Eagle's medium supplemented with 10% fetal bovine serum (FBS) and 1% penicillin/streptomycin (Thermo Fisher). For transfection, 300,000 U2OS cells were nucleofected (SE, DN-100) using the 4D nucleofector (Lonza) with a total of 750 ng of plasmid DNA encoding the designer nucleases. Cells were allowed to recover in complete medium in 24-well plates for 3 days before genotyping. Peripheral blood mononuclear cells (PBMCs) were cultured with T cell medium, composed of RPMI medium supplemented with 10% FBS, 1% penicillin/streptomycin, and 10 mM HEPES buffer (all Thermo Fisher). T cells were activated using soluble anti-CD2/3/28 (Stem Cell Technology) for 3 days in T cell medium supplemented with 100 U/mL of human IL-2 (hIL-2), 50 U/mL of hIL-15, and 25 U/mL of hIL-7 (Miltenyi Biotec). 1×10^6 T cells were nucleofected (P3, EO-115) using the 4D nucleofector (Lonza) with either 3 μg of each TALEN mRNA or 3 μg of Cas9 protein complexed with 100 pmol of gRNA for 10 min at room temperature (RT). After nucleofection, T cells recovered in 96-well plates (Corning, U bottom). The first day post-nucleofection, the IL-2 concentration was increased to 1,000 U/mL. T cells were split every 3 days to densities of 2×10^6 cells/mL for 7 days before phenotypic and genotypic analyses. Genotyping of *TRAC*-edited cells was performed using the T7 endonuclease I (T7E1, NEB) assay as previously described.[32] In brief, edited T cells were harvested at the end of the expansion phase or from the post-depleted product. Genomic DNA was extracted using QIAamp DNA mini kit (-QIAGEN). An amplicon encompassing the TALEN target site in the *TRAC* locus was generated by PCR using primer pair (5′-taaagcat-gagaccgtgact-3′ and 5′-tagacatcattgaccagagc-3′), purified using QIAquick PCR purification kit (QIAGEN), and subjected to T7E1. To determine the extent of indel frequency, the band intensities were quantified using ImageJ software (https://imagej.nih.gov/ij/index.html).

On-target and off-target analysis

Potential off-target sites for the TALEN pair were predicted using the online tool PROGNOS (TALEN v2.0).[33] Applied parameters allowed for up to 6 mismatches for each TALEN target half-site, a spacing distance between the TALEN monomers of 10 to 25 bp, considering both hetero- and homodimers. The top-ranking off-targets were considered for targeted Amp-seq. Potential off-targets of the CRISPR-Cas9 nucleases were predicted using the online tool COSMID.[34] The query allowed for hits with up to 3 mismatches or 1-bp deletion/insertion with up to 2 mismatches. The top 20 scoring off-target sites were analyzed by next-generation targeted Amp-seq. PCR was performed on genomic DNA isolated from gene-edited cells to generate amplicons spanning the *TRAC* on-target site and the predicted potential off-target sites. The resulting amplicons were processed to a DNA library (NEBNext, Ultra II DNA library prep kit for Illumina, NEB #E7645L), quantified using digital droplet PCR (ddPCR) library quantification with Illumina TruSeq (Bio-Rad #186-3040,) and sequenced on an Illumina MiSeq using a MiSeq reagent kit v2, 500 cycles (Illumina, MS-102-2003). The paired-end reads were analyzed using CRISPResso2 tool.[35] Obtained p values were adjusted using the Benjamin-Hochberg correction method as described previously.[36]

Optimization of the electroporation conditions in small scale

PBMCs-derived T cells or CD4/8 selected T cells were thawed and recovered for 24 h with TexMACS GMP medium supplemented with 12.5 ng/mL of recombinant hIL-7 and 12.5 ng/mL of recombinant hIL-15. On the same day or 24 h later, T cells were activated with MACS GMP T cell TransAct (Miltenyi Biotec), according to the manufacturer's instructions, for 3 days prior to electroporation. For TALEN mRNA transfer, 1×10^6 activated T cells were electroporated with 7.5 μg of each TALEN mRNA, unless stated otherwise, in a final volume of 50 μL CliniMACS electroporation buffer (Miltenyi Biotec) in the test cuvette adaptor using the electroporation settings listed in Table 1.

Automated generation of TCRα/β-free CAR T cells

TCRα/β-free CAR T cells were manufactured using the newly developed, automated, and closed T cell engineering (TCE) process on the CliniMACS Prodigy platform during the entire process. Buffy coats or leukapheresates of healthy donors were obtained from the Blood Donation Center of the Medical Center - University of Freiburg or from the German Red Cross Blood Donation Service Baden-Württemberg - Hessen (obtained with donor consent after ethical review). The manufacturing process is composed of 4 stages: (1) process parameters were entered in the activity matrix. For CD4/CD8 T cell selection and activation, buffy coats were evaluated in terms of white blood cell (WBC) concentration and frequencies of CD4-positive and CD8-positive cells prior to the selection step. Then, buffy coats were connected to the tubing set, T cells labeled and magnetically isolated using CliniMACS CD4 reagent and CliniMACS CD8 reagent

(Miltenyi Biotec). Following T cell selection, QC samples were taken to evaluate the purity of the T cells post-selection and the total T cell number. 2×10^8 selected T cells were automatically transferred to the cultivation chamber and cultivated in TexMACS GMP medium supplemented with 12.5 ng/mL of recombinant human IL-7 and recombinant human IL-15 (Miltenyi Biotec), respectively. Subsequently, T cells were activated using the contents of one vial of the MACS GMP T cell TransAct (Miltenyi Biotec). (2) For TCE, 1 day post-activation, T cells were transduced with lentiviral particles (Miltenyi Biotec) coding for the CAR transgene by connection of the bag to the Prodigy via sterile welding. At day 3 post-activation, cells were rebuffered in CliniMACS electroporation buffer (Miltenyi Biotec); 1.1–1.4 mg of each TALEN mRNA were transferred to the nucleic acid bag, and electroporation was started using setting 3 described in Table 1. (3) For expansion of edited T cells following electroporation, cells were transferred back to the cultivation chamber and recovered in 66 mL TexMACS media supplemented with IL-7 and IL-15, and static culture was performed for 24 h and then switched back to agitated modus. Afterward, cells were expanded for 9–13 days. Medium was changed automatically via centrifugation and automatic media feed every other day. Since T cell stimulation reagent might influence the TCE process, we chose to base our work on a previously described manufacturing process,[17–19] in which MACS GMP T cell TransAct was shown to yield good T cell stimulation and expansion as well as compatibility with closed manufacturing systems, such as the CliniMACS Prodigy, where excess reagent can be easily removed by medium wash not requiring magnetic bead removal in the final cellular product. (4) For TCRα/β depletion and final formulation, at the end of the expansion phase, total T cells were evaluated and the fraction of TCRα/β-negative cells was assessed. For the depletion, we processed only half of the culture in order to keep some cells for QC and functional test from the pre-depletion fraction. Cells were harvested in CliniMACS PBS/EDTA buffer (Miltenyi Biotec) supplemented with 0.5% bovine serum albumin. The new tubing set TS320 was mounted. During the TCRα/β depletion process, the cells were labeled and magnetically selected with CliniMACS TCRα/β-biotin and CliniMACS anti-biotin reagents (Miltenyi Biotec) and finally formulated in CliniMACS PBS/EDTA buffer (Miltenyi Biotec). The final cell products were either directly used for functional analysis or frozen until further use. The purity of the TCRα/β depletion step was assessed by flow cytometry, and the TCRα/β-free CAR T cells were counted and frozen.

Antibodies and flow cytometry

To determine cellular composition and CD4/CD8 ratios, we stained the cells with anti-CD45-vioblue, CD56-phycoerythrin (PE), CD3-fluorescein isothiocyanate (FITC), CD4-viogreen, CD8-APC/Vio770, CD20-PE/Vio770, and CD14-allophycocyanin (APC) for 10 min at 4–8°C. To determine the CAR expression, cells were stained using the CD19-CAR detection reagent (Miltenyi Biotec) (10–20 min at RT), then cells were washed twice with fluorescence-activated cell sorting (FACS) buffer and stained with anti-biotin-PE (10–20 min at 4°C–8°C). For T cell phenotype, cells were stained with the CD19-CAR detection reagent, CD3-APC, CD62L-Bv421 (BD Biosciences), and CD45RA-FITC (BioLegend) antibodies as described above for the CAR staining. To evaluate the knockout efficiencies, cells were stained with TCRα/β-PE or with CD3-APC antibodies (30–40 min at 4°C–8°C). To characterize the CD3-positive cells, cells were stained with CD3-APC and CD56-PE to identify NK and NK T cells (NKT) cells, respectively. Anti-CD3-APC, pan-TCRαβ-PE, and pan-TCRγ/δ-vioblue antibodies used to distinguish TCRαβ and TCRγ/δ T cells (40 min at 4°C–8°C). To determine cell viability, DAPI or 7-amino-actinomycin (7-AAD) was added to the cells to discriminate dead cells using flow cytometer or NucleoCounter-NC-250 (Chemometec). Flow cytometric measurements were performed using the MACSQuant analyzer 10 (Miltenyi Biotec), FACS Canto II, or Accuri (BD Biosciences). Data were analyzed using either MACSQuantify 2.8 (Miltenyi Biotec) or FlowJo 10 (BD Biosciences). All antibodies, unless mentioned otherwise, were from Miltenyi Biotec.

Cytotoxicity and multiplex cytokine release assay

Cytotoxicity assay was performed as previously described.[19] In brief, effector T cells were co-cultured with CD19-positive/GFP-positive Jeko-1 cells, CD19-positive/GFP-positive Nalm6 cells, or with CD19-negative K562 cells labeled with CellTrace violet (Thermo Fisher) at the indicated E:T ratios in round-bottom 96-well plates in a total volume of 200 μL TexMACS medium without cytokines for 20 h. Specific lysis was determined based on the number of viable GFP-positive or CellTrace violet-labeled target cells. As a control, untransduced T cells were used and cultured with the same E:T ratio. Cytotoxicity was calculated according to the following equation: $([\#\text{ of events of }_{T\text{ only}} - \#\text{ of events }_{T+E}]/[\#\text{ of events }_{T\text{ only}}]) \times 100\%$; E, effector cells; T, target cells. To determine cytokine release of the 1:1 E:T ratio, 100 μL of the supernatants were harvested 20 h post-co-culture and kept at −20°C until analyzed. The cytokines were quantified using the human MACSPlex cytokine 12 kit (Miltenyi Biotec) according to the manufacturer's instructions.

Statistical analysis

Statistical significance in all experiments was determined using the unpaired Student's t test (GraphPad Prism).

SUPPLEMENTAL INFORMATION

Supplemental Information can be found online at https://doi.org/10.1016/j.omtm.2020.12.008.

ACKNOWLEDGMENTS

The authors thank Julia Dzionek and her team for developing the TCRα/β depletion process, Ruba Hammad and Beate vom Hövel for their technical assistance, and the Lighthouse Core Facility and the Blood Donation Center, both Medical Center - University of Freiburg, for help with flow cytometry and for support with buffy coats. This work was supported by grants from the Horizon 2020 Programme of the European Commission (CARAT-667980 to T.C., S.W., A.K., and T.S.) and the German Federal Ministry of Education and Research (IFB-01EO1303 to T.C., T.I.C., and C.M.).

AUTHOR CONTRIBUTIONS

T.C., A.K., and T.I.C. conceived and supervised the study; J.A., D.L, M.R., C.B., J.R., and S.L. carried out the cell-based experiments; M.H., K.O.C., and S.A.H. performed NGS; C.M. designed the *TRAC* TALEN; S.W. generated TALEN mRNA; S.S., S.W., and D.L. developed the T cell engineering process; J.A., D.L., T.I.C., M.R., T.S., A.K., and T.C. planned the experiments and interpreted the data; J.A., D.L., M.R, T.S., A.K., T.I.C., and T.C. wrote the manuscript.

DECLARATION OF INTERESTS

D.L., S.S., S.W., C.B., S.L., T.S., and A.K. are employees of Miltenyi Biotec. T.C. and T.I.C. have sponsored research collaborations with Miltenyi Biotech and Cellectis.

REFERENCES

1. Mussolino, C., Alzubi, J., Pennucci, V., Turchiano, G., and Cathomen, T. (2017). Genome and Epigenome Editing to Treat Disorders of the Hematopoietic System. Hum. Gene Ther. *28*, 1105–1115.
2. Salmikangas, P., Kinsella, N., and Chamberlain, P. (2018). Chimeric Antigen Receptor T-Cells (CAR T-Cells) for Cancer Immunotherapy - Moving Target for Industry? Pharm. Res. *35*, 152.
3. Qasim, W., Zhan, H., Samarasinghe, S., Adams, S., Amrolia, P., Stafford, S., Butler, K., Rivat, C., Wright, G., Somana, K., et al. (2017). Molecular remission of infant B-ALL after infusion of universal TALEN gene-edited CAR T cells. Sci. Transl. Med. *9*, eaaj2013.
4. MacLeod, D.T., Antony, J., Martin, A.J., Moser, R.J., Hekele, A., Wetzel, K.J., Brown, A.E., Triggiano, M.A., Hux, J.A., Pham, C.D., et al. (2017). Integration of a CD19 CAR into the TCR Alpha Chain Locus Streamlines Production of Allogeneic Gene-Edited CAR T Cells. Mol. Ther. *25*, 949–961.
5. Eyquem, J., Mansilla-Soto, J., Giavridis, T., van der Stegen, S.J., Hamieh, M., Cunanan, K.M., Odak, A., Gönen, M., and Sadelain, M. (2017). Targeting a CAR to the TRAC locus with CRISPR/Cas9 enhances tumour rejection. Nature *543*, 113–117.

[6]. Wiebking, V., Lee, C.M., Mostrel, N., Lahiri, P., Bak, R., Bao, G., et al. (2020). Genome editing of donor-derived T-cells to generate allogenic chimeric antigen receptor-modified T cells: Optimizing αβ T cell-depleted haploidentical hematopoietic stem cell transplantation. Haematologica *105*, 233882.

7. Stenger, D., Stief, T.A., Kaeuferle, T., Willier, S., Rataj, F., Schober, K., Vick, B., Lotfi, R., Wagner, B., Grünewald, T.G.P., et al. (2020). Endogenous TCR promotes in vivo persistence of CD19-CAR-T cells compared to a CRISPR/Cas9-mediated TCR knockout CAR. Blood *136*, 1407–1418.
8. Juillerat, A., Tkach, D., Yang, M., Boyne, A., Valton, J., Poirot, L., and Duchateau, P. (2020). Straightforward Generation of Ultrapure Off-the-Shelf Allogeneic CAR-T Cells. Front. Bioeng. Biotechnol. *8*, 678.
9. Provasi, E., Genovese, P., Lombardo, A., Magnani, Z., Liu, P.Q., Reik, A., Chu, V., Paschon, D.E., Zhang, L., Kuball, J., et al. (2012). Editing T cell specificity towards leukemia by zinc finger nucleases and lentiviral gene transfer. Nat. Med. *18*, 807–815.
10. Mastaglio, S., Genovese, P., Magnani, Z., Ruggiero, E., Landoni, E., Camisa, B., Schiroli, G., Provasi, E., Lombardo, A., Reik, A., et al. (2017). NY-ESO-1 TCR single edited stem and central memory T cells to treat multiple myeloma without graft-versus-host disease. Blood *130*, 606–618.
11. Poirot, L., Philip, B., Schiffer-Mannioui, C., Le Clerre, D., Chion-Sotinel, I., Derniame, S., Potrel, P., Bas, C., Lemaire, L., Galetto, R., et al. (2015). Multiplex Genome-Edited T-cell Manufacturing Platform for “Off-the-Shelf” Adoptive T-cell Immunotherapies. Cancer Res. *75*, 3853–3864.
12. Cornu, T.I., Mussolino, C., and Cathomen, T. (2017). Refining strategies to translate genome editing to the clinic. Nat. Med. *23*, 415–423.
13. Qasim, W., Zhan, H., Samarasinghe, S., Adams, S., Amrolia, P., Stafford, S., Butler, K., Rivat, C., Wright, G., Somana, K., et al. (2017). Molecular remission of infant B-ALL after infusion of universal TALEN gene-edited CAR T cells. Sci. Transl. Med. *9*, eaaj2013.
14. Straetemans, T., Kierkels, G.J.J., Doorn, R., Jansen, K., Heijhuurs, S., Dos Santos, J.M., van Muyden, A.D.D., Vie, H., Clemenceau, B., Raymakers, R., et al. (2018). GMP-Grade Manufacturing of T Cells Engineered to Express a Defined γδTCR. Front. Immunol. *9*, 1062.
15. Krug, C., Wiesinger, M., Abken, H., Schuler-Thurner, B., Schuler, G., Dörrie, J., and Schaft, N. (2014). A GMP-compliant protocol to expand and transfect cancer patient T cells with mRNA encoding a tumor-specific chimeric antigen receptor. Cancer Immunol. Immunother. *63*, 999–1008.
16. Wiesinger, M., März, J., Kummer, M., Schuler, G., Dörrie, J., Schuler-Thurner, B., and Schaft, N. (2019). Clinical-Scale Production of CAR-T Cells for the Treatment of Melanoma Patients by mRNA Transfection of a CSPG4-Specific CAR under Full GMP Compliance. Cancers (Basel) *11*, 1198.
17. Priesner, C., Aleksandrova, K., Esser, R., Mockel-Tenbrinck, N., Leise, J., Drechsel, K., Marburger, M., Quaiser, A., Goudeva, L., Arseniev, L., et al. (2016). Automated Enrichment, Transduction, and Expansion of Clinical-Scale $CD62L^+$ T Cells for Manufacturing of Gene Therapy Medicinal Products. Hum. Gene Ther. *27*, 860–869.
18. Mock, U., Nickolay, L., Philip, B., Cheung, G.W., Zhan, H., Johnston, I.C.D., Kaiser, A.D., Peggs, K., Pule, M., Thrasher, A.J., and Qasim, W. (2016). Automated manufacturing of chimeric antigen receptor T cells for adoptive immunotherapy using CliniMACS prodigy. Cytotherapy *18*, 1002–1011.
19. Lock, D., Mockel-Tenbrinck, N., Drechsel, K., Barth, C., Mauer, D., Schaser, T., Kolbe, C., Al Rawashdeh, W., Brauner, J., Hardt, O., et al. (2017). Automated Manufacturing of Potent CD20-Directed Chimeric Antigen Receptor T Cells for Clinical Use. Hum. Gene Ther. *28*, 914–925.
20. Jackson, Z., Roe, A., Sharma, A.A., Lopes, F.B.T.P., Talla, A., Kleinsorge-Block, S., Zamborsky, K., Schiavone, J., Manjappa, S., Schauner, R., et al. (2020). Automated Manufacture of Autologous CD19 CAR-T Cells for Treatment of Non-hodgkin Lymphoma. Front. Immunol. *11*, 1941.
21. Kaiser, A.D., Assenmacher, M., Schröder, B., Meyer, M., Orentas, R., Bethke, U., and Dropulic, B. (2015). Towards a commercial process for the manufacture of genetically modified T cells for therapy. Cancer Gene Ther. *22*, 72–78.
22. de Witte, M.A., Kuball, J., and Miller, J.S. (2017). NK Cells and γδT Cells for Relapse Protection After Allogeneic Hematopoietic Cell Transplantation (HCT). Curr. Stem Cell Rep. *3*, 301–311.
23. Ruella, M., Xu, J., Barrett, D.M., Fraietta, J.A., Reich, T.J., Ambrose, D.E., Klichinsky, M., Shestova, O., Patel, P.R., Kulikovskaya, I., et al. (2018). Induction of resistance to chimeric antigen receptor T cell therapy by transduction of a single leukemic B cell. Nat. Med. *24*, 1499–1503.
24. Chen, P.H., Lipschitz, M., Weirather, J.L., Jacobson, C., Armand, P., Wright, K., Hodi, F.S., Roberts, Z.J., Sievers, S.A., Rossi, J., et al. (2020). Activation of CAR and non-CAR T cells within the tumor microenvironment following CAR T cell therapy. JCI Insight *5*, e134612.
25. Monjezi, R., Miskey, C., Gogishvili, T., Schleef, M., Schmeer, M., Einsele, H., Ivics, Z., and Hudecek, M. (2017). Enhanced CAR T-cell engineering using non-viral Sleeping Beauty transposition from minicircle vectors. Leukemia *31*, 186–194.
26. Cooper, M.L., Choi, J., Staser, K., Ritchey, J.K., Devenport, J.M., Eckardt, K., Rettig, M.P., Wang, B., Eissenberg, L.G., Ghobadi, A., et al. (2018). An “off-the-shelf” fratricide-resistant CAR-T for the treatment of T cell hematologic malignancies. Leukemia *32*, 1970–1983.
27. John, L.B., Kershaw, M.H., and Darcy, P.K. (2013). Blockade of PD-1 immunosuppression boosts CAR T-cell therapy. OncoImmunology *2*, e26286.
28. Sachdeva, M., Busser, B.W., Temburni, S., Jahangiri, B., Gautron, A.S., Maréchal, A., Juillerat, A., Williams, A., Depil, S., Duchateau, P., et al. (2019). Repurposing endogenous immune pathways to tailor and control chimeric antigen receptor T cell functionality. Nat. Commun. *10*, 5100.
29. Ren, J., Liu, X., Fang, C., Jiang, S., June, C.H., and Zhao, Y. (2017). Multiplex Genome Editing to Generate Universal CAR T Cells Resistant to PD1 Inhibition. Clin. Cancer Res. *23*, 2255–2266.
30. Mussolino, C., Morbitzer, R., Lütge, F., Dannemann, N., Lahaye, T., and Cathomen, T. (2011). A novel TALE nuclease scaffold enables high genome editing activity in combination with low toxicity. Nucleic Acids Res. *39*, 9283–9293.

31. Labun, K., Montague, T.G., Gagnon, J.A., Thyme, S.B., and Valen, E. (2016). CHOPCHOP v2: a web tool for the next generation of CRISPR genome engineering. Nucleic Acids Res. *44* (W1), W272–W276.

32. Mussolino, C., Alzubi, J., Fine, E.J., Morbitzer, R., Cradick, T.J., Lahaye, T., Bao, G., and Cathomen, T. (2014). TALENs facilitate targeted genome editing in human cells with high specificity and low cytotoxicity. Nucleic Acids Res. *42*, 6762–6773.

33. Fine, E.J., Cradick, T.J., Zhao, C.L., Lin, Y., and Bao, G. (2014). An online bioinformatics tool predicts zinc finger and TALE nuclease off-target cleavage. Nucleic Acids Res. *42*, e42.

34. Cradick, T.J., Qiu, P., Lee, C.M., Fine, E.J., and Bao, G. (2014). COSMID: A Web-based Tool for Identifying and Validating CRISPR/Cas Off-target Sites. Mol. Ther. Nucleic Acids *3*, e214.

35. Clement, K., Rees, H., Canver, M.C., Gehrke, J.M., Farouni, R., Hsu, J.Y., Cole, M.A., Liu, D.R., Joung, J.K., Bauer, D.E., and Pinello, L. (2019). CRISPResso2 provides accurate and rapid genome editing sequence analysis. Nat. Biotechnol. *37*, 224–226.

36. Benjamini, Y., and Hochberg, Y. (1995). Controlling the False Discovery Rate - a Practical and Powerful Approach to Multiple Testing. J. R. Statist. Soc. B *57*, 289–300.

OMTM, Volume *20*

Supplemental Information

Automated generation of gene-edited CAR T cells at clinical scale

Jamal Alzubi, Dominik Lock, Manuel Rhiel, Sabrina Schmitz, Stefan Wild, Claudio Mussolino, Markus Hildenbeutel, Caroline Brandes, Julia Rositzka, Simon Lennartz, Simone A. Haas, Kay O. Chmielewski, Thomas Schaser, Andrew Kaiser, Toni Cathomen, and Tatjana I. Cornu

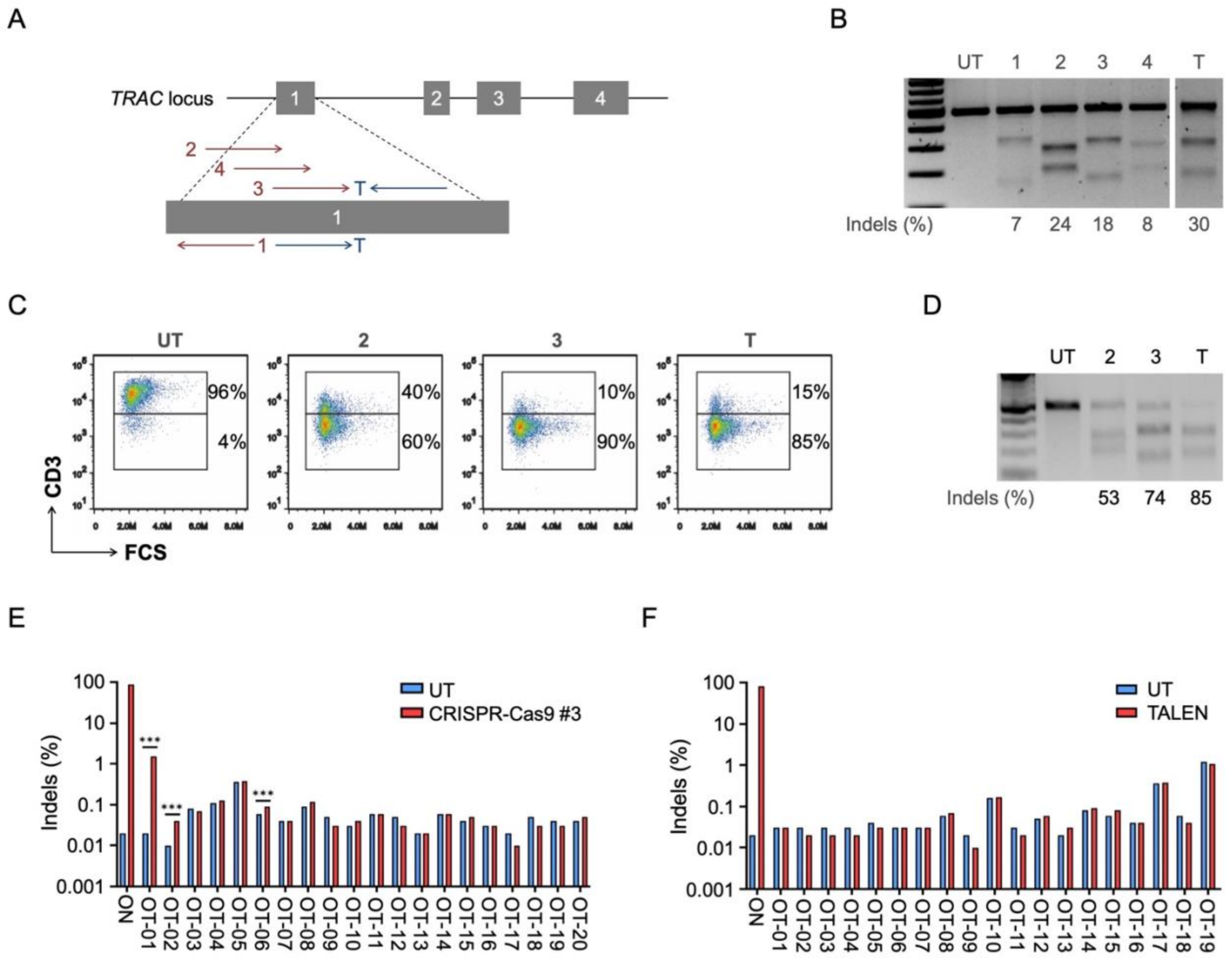

Figure S1. Characterization of *TRAC*-targeting designer nucleases. (A) Target sites in *TRAC* locus. Shown are the target sites in *TRAC* exon 1 of all designer nucleases. Red arrows denote CRISPR-Cas9 target sites #1-4, blue arrows represent binding half-sites of the TALEN (T). **(B)** Activity of designer nucleases in U2OS cells. U2OS cells transfected with designer nuclease expression plasmids (#1-4, T) were subjected to T7E1 assay. Percentages of cleaved products are indicated. Shown are results from one experiment. **(C)** Phenotyping of TALEN-edited T cells. Primary T cells nucleofected with either CRISPR-Cas9 ribonucleoprotein (RNP) complexes (#2-3) or TALEN-encoding mRNA (T) were subjected to flow cytometry. Shown are fractions of CD3-positive or CD3-negative cells harvested 7 days post-nucleofection. **(D)** Genotyping of TALEN-edited T cells. Primary T cells nucleofected with RNPs (#2-3) or TALEN-mRNA (T) were subjected to T7E1 assay. Percentages of cleaved products are indicated. Shown are results from one experiment. **(E-F)** Specificity profiles of *TRAC*-targeting designer nucleases. Primary T cells nucleofected with RNP (E) or TALEN mRNA (F) were subjected to targeted amplicon sequencing. Indel frequencies at the *TRAC* on-target site (ON) and potential off-target (OT) sites are indicated in comparison to untreated (UT) T cells. Shown are results from one donor. *** $p \leq 0.001$; Indels, insertion/deletion mutations.

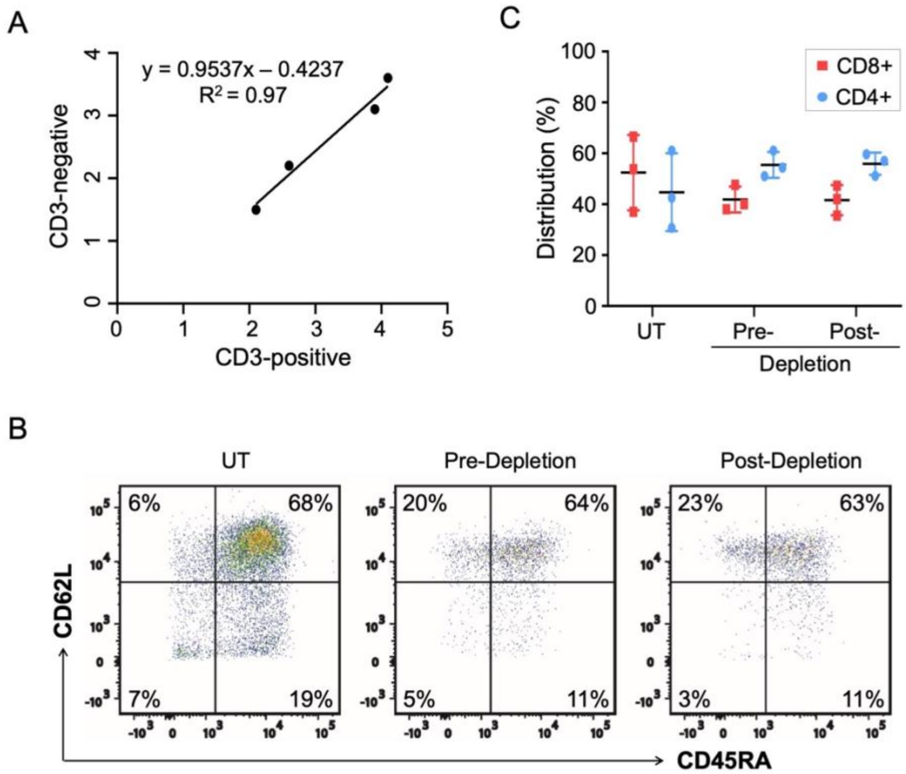

Figure S2. Quality assessment of *TRAC*-edited CAR T cells. (A) Comparison of cell division rates. Shown are the numbers of cell divisions for CD3-negative vs. CD3-positive cells determined at different time points during the manufacturing process (days 6, 7, 10, 12). Data were collected from all three manufacturing runs. **(B)** T cell phenotype analysis. T cell subsets were determined by assessing the expression of CD62L and CD45RA by flow cytometry. Edited CAR T cells were pre-gated for CD3 expression for UT, or CAR expression for the fraction of pre-depleted and post-depleted CAR T cells (not shown). Representative plots of one run are shown. **(C)** CD4 to CD8 ratio. Engineered T cells of three independent runs were harvested at indicated processing steps and CD4:CD8 ratio determined by flow cytometry.

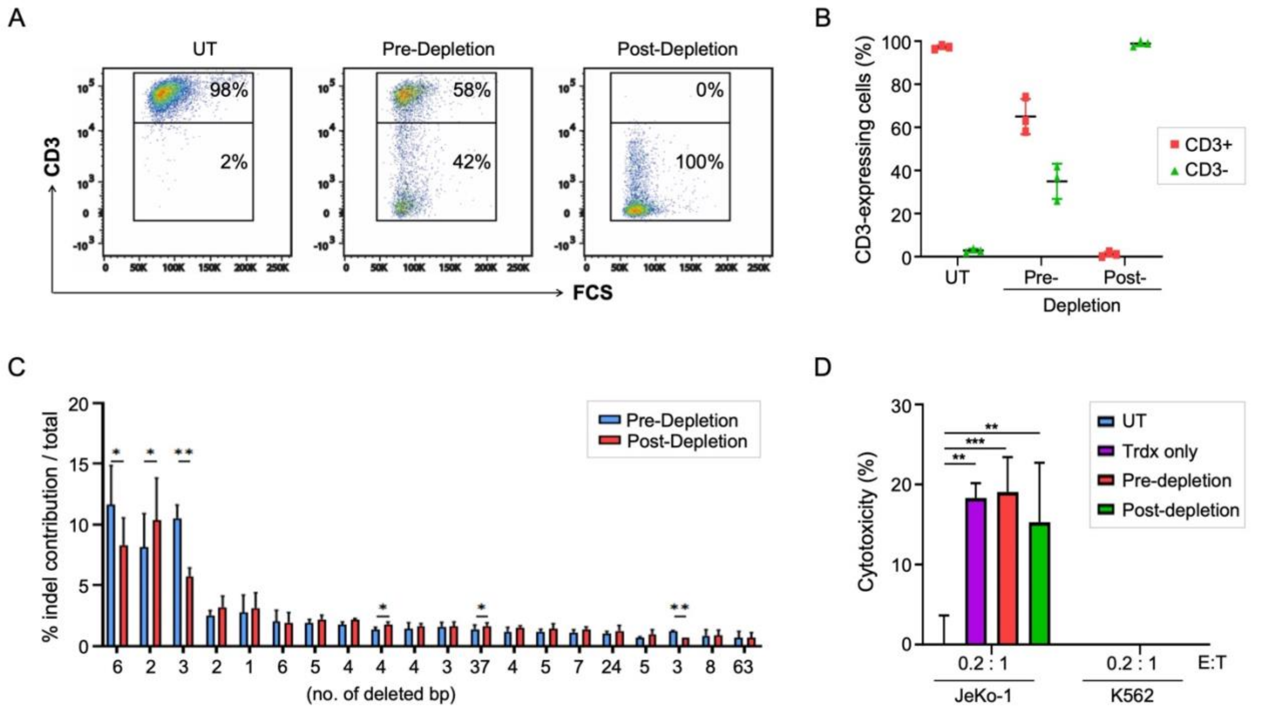

Figure S3. Characterization of *TRAC*-edited CAR T cells. (A) Phenotypic analysis of *TRAC* knockout. Engineered T cells were harvested at indicated processing steps. *TRAC* disruption was evaluated by flow cytometry analysis of CD3 expression. Representative plots of one run are shown. **(B)** Synopsis of three manufacturing runs. Shown are the fractions of CD3-positive and CD3-negative cells at indicated processing steps. **(C)** Indel distribution. Engineered T cells of pre and post-depletion steps were subjected to targeted amplicon sequencing of the *TRAC* locus. Shown are results of three manufacturing runs. Numbers on x-axis indicate the number of deleted bp. Indel, insertion/deletion; UT, untreated T cells. **(D)** Cytolytic activity. CAR T cells from indicated groups were co-cultured at an E:T ratio of 0.2:1 with CD19-positive JeKo-1 cells or CD19-negative K562 cells. Cytotoxicity was determined by flow cytometry. Shown are results of two or three independent runs, each in duplicate. UT, untreated T cells; Trdx only, CAR T cells retrieved directly after lentiviral transduction; Pre-depletion, CAR T cells harvested before TCRα/β depletion; Post-depletion, CAR T cells harvested after TCRα/β depletion.

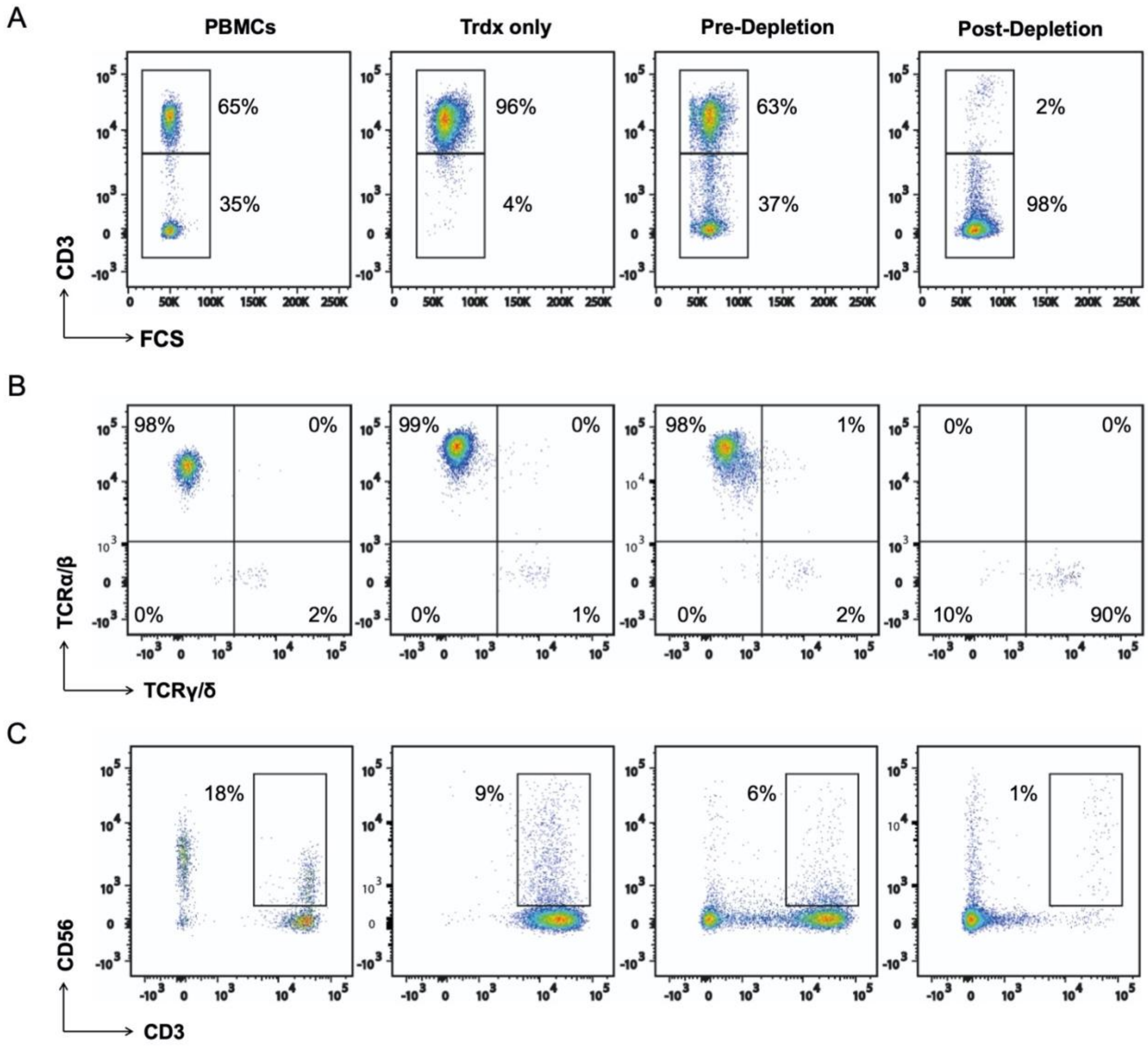

Figure S4. Characterization of final CAR T cell product. Engineered T cells were harvested at indicated processing steps and characterized in terms of **(A)** CD3 expression, **(B)** TCRα/β and TCRγ/δ expression, and **(C)** CD56 expression to identify the presence of NK and NKT cells. Shown are results of one representative run. PBMCs, peripheral blood mononuclear cells; Trdx only, T cells transduced with CD19-CAR encoding lentiviral particles only.
